# Impact of urgent resuscitative surgery for life-threatening torso trauma

**DOI:** 10.1007/s00595-016-1451-0

**Published:** 2016-11-25

**Authors:** Hisashi Matsumoto, Yoshiaki Hara, Takanori Yagi, Nobuyuki Saito, Kazuki Mashiko, Hiroaki Iida, Tomokazu Motomura, Fumihiko Nakayama, Kazuhiro Okada, Hiroshi Yasumatsu, Taigo Sakamoto, Takao Seo, Yusuke Konda, You Hattori, Hiroyuki Yokota

**Affiliations:** 1 Shock and Trauma Centre, Nippon Medical School Chiba Hokusoh Hospital, 1715, Kamakari, Inzai, Chiba Prefecture 270-1694 Japan; 2 Department of Emergency and Critical Care Medicine, Nippon Medical School, Tokyo, Japan

**Keywords:** Trauma resuscitation, Hemostatic operation, Resuscitative thoracotomy, Damage control surgery, Emergency department

## Abstract

**Purpose:**

This study investigated the advantages of performing urgent resuscitative surgery (URS) in the emergency department (ED); namely, our URS policy, to avoid a delay in hemorrhage control for patients with severe torso trauma and unstable vital signs.

**Methods:**

We divided 264 eligible cases into a URS group (*n* = 97) and a non-URS group (*n* = 167) to compare, retrospectively, the observed survival rate with the predicted survival using the Trauma and Injury Severity Score (TRISS).

**Results:**

While the revised trauma score and the injury severity score were significantly lower in the URS group than in the non-URS group, the observed survival rate was significantly higher than the predicted rate in the URS (48.5 vs. 40.2%; *p* = 0.038). URS group patients with a systolic blood pressure (SBP) <90 mmHg and a Glasgow coma scale (GCS) score of ≥9 had significantly higher observed survival rates than predicted survival rates (0.433 vs. 0.309, *p* = 0.008), (0.795 vs. 0.681, *p* = 0.004). The implementation of damage control surgery (DCS) was found to be a significant predictor of survival (OR 5.23, 95% CI 0.113–0.526, *p* < 0.010).

**Conclusion:**

The best indications for the URS policy are an SBP <90 mmHg, a GCS ≥9 on ED arrival, and/or the need for DCS. By implementing our URS policy, satisfactory survival of patients requiring immediate hemostatic surgery was achieved.

## Introduction

Saving the life of a patient with severe torso trauma and ensuing hemorrhagic shock necessitating hemostatic surgery present serious challenges. Such injuries include thoracic cage or thoracic organ trauma, abdominal organ trauma, or unstable pelvic fracture. If the injured patient suffers an impending cardiac arrest at the scene of injury, they may not arrive at the emergency department (ED) in time [1]. Even if they are brought to the ED, there is rarely time to transport them to the operating room (OR) before cardiac arrest occurs [2, 3]. Moreover, the patient may suffer coagulopathy caused by various factors while waiting for preparation of the OR.

Surgical hemostatic control for severe trauma should be performed in a fully equipped OR; however, vital signs can deteriorate quickly during transportation to the OR, resulting in irreparable damage [2, 4, 5]. Furthermore, the OR may not be immediately available because of ongoing scheduled operations, and any delay in hemostasis increases the risk of mortality for patients with severe trauma. Thus, it is crucial to establish appropriate criteria for deciding where to perform hemostatic surgery, regardless of special layouts and/or the facility design of the hospital.

Considering the fact that the early death after arrival to the hospital may be averted by hemostatic surgery being performed immediately, our facility has implemented a policy for urgent resuscitative surgery (URS) in the ED to avoid a delay in hemorrhage control and avert potentially higher mortality of patients with severe torso trauma and unstable vital signs. This policy consists of the following three steps to prevent cardiac arrest from hemorrhagic shock: immediate surgical bleeding control, the early decision about damage control surgery (DCS) [6], and non-operative resuscitation [7–9].

Over the last 15 years, we have developed a trauma system in the east Kanto area of Japan, which involves training emergency medical technicians (EMT) [10] and establishing a helicopter emergency medical service (HEMS) [11]. To further improve the outcomes of these patients, it is important that we confirm the appropriateness of our hospital’s procedure for managing severe trauma. This report studies the advantages of urgent resuscitative hemostatic surgery in the ED (URS policy) to confirm the effectiveness of our treatment practices for patients with severe torso trauma.

## Methods

### Study design

This was a retrospective study of patients who underwent urgent surgical intervention between January, 2007 and December, 2014, to control massive bleeding at our facility, which corresponds to a Level 1 trauma centre in the US. We collected the following data from the clinical records: demographics, injury mechanism, vital signs, reason for surgery, surgical procedure, total resuscitation volume before surgery, total transfused blood products within 24 h (packed red blood cells: PRBC, fresh frozen plasma: FFP), time between the hospital arrival to the operation, revised trauma score (RTS) and injury severity score (ISS), and then probability of survival (Ps) based on the trauma and injury severity score (TRISS). Patients who suffered cardiac arrest on ED admission, or had a maximum Abbreviated Injury Scale score (AIS) = 6, or patients with missing data, were excluded from the analysis.

When a patient had an impending cardiac arrest with suspected torso trauma or a massive hemothorax or hemoperitoneum with hemorrhagic shock, we applied the URS policy for trauma resuscitation, that is, immediate surgical bleeding control with non-surgical resuscitation in the ED (damage control resuscitation: DCR) [7–9] (Table 1). Surgical bleeding control was defined as resuscitative thoracotomy for bleeding control of life-threatening chest injuries or for aortic cross clamping [12–15], celiotomy to control hemorrhage from the abdominal organs, or retroperitoneal packing for an unstable pelvic fracture such as Tile C. In addition, DCS as a hemostatic strategy was performed for patients with chest injury as well as subdiaphragmatic injury under the early DCS decision criteria [6].

**Table 1:** Our damage control resuscitation (DCR) protocol based on the seven tactics in the Emergency Department (ED) with simultaneous hemostatic surgery

1.	Massive transfusion protocol
2.	Permissive hypotension before hemostasis (SBP 70–90 mmHg)
3.	Vasoconstrictor (noradrenaline) administration for persistent shock
4.	Aggressive calcium supplementation (target: >1.0 mmol/dL)
5.	Early administration of tranexamic acid
6.	Warming the body during resuscitation
7.	Evaluation of body fluid balance and maintenance of hemodynamics in the ICU

*DCR* damage control resuscitation, *ED* emergency department, *SBP* systolic blood pressure, *ICU* intensive care unit

Whether to apply the URS policy was initially decided based on prehospital information from the EMT personnel and activated secondarily at the discretion of the emergency physician in the ED, based on the patient’s vital signs and simple image examinations, including portable chest and pelvic radiography, and focused assessment sonography for trauma (FAST). To evaluate our policy, we divided the eligible patients into two groups according to whether URS was performed (URS group) or not (non-URS group).

### Evaluation of survival rate

The observed survival rate in this cohort was compared with the predicted survival rate, using the TRISS methodology [16]. The difference between the observed survival rate and the TRISS probability of survival was examined using the following formula established by Rhodes et al. [5]:

$${\text{TRAIS}} = 1\left( {\text{alive}} \right){\text{or }}0\left( {\text{dead}} \right) - {\text{TRISS probability of survival}} .$$

Rhodes’ et al. stated that “a TRISS adjusted increment in survivability (TRAIS) was calculated for each patient. Each patient was scored 1 or 0 according to whether the patient survived or died. The TRAIS ranges from −1 for a patient who was predicted to survive but died to 1 for a patient who was predicted to die but survived. Thus, an average TRAIS = 0 implies a survival rate equal to the TRISS-base average. An average TRAIS <0 implies a survival rate worse than average, and an average TRAIS >0 implies one greater than average.” (quoted from Ref. [5])

### Data analysis

Data are expressed as medians [IQR] except for the mean of TRISS and TRAIS. The chi-square test for categorical variables and Mann–Whitney *U* test for intergroup comparisons between two groups were performed, and one-sample *t* test using the TRAIS values was used for the evaluation of survival results under the guidance of three statisticians. The predicted mortality related to each surgical procedure was calculated using logistic regression formulae. Statistical analyses were performed by STATVIEW and Microsoft Excel software, and *p* < 0.05 was considered significant.

## Results

### Study population

Of a total 434 consecutive patients who underwent urgent hemostatic surgery during the study period, 264 were eligible for this analysis, after excluding 164 who had suffered cardiac arrest on arrival to the ED, 5 who had a maximum AIS = 6, and 1 with incomplete data. Table 2 summarizes the baseline characteristics of the eligible patients. The median age of the study cohort was 50 (33–65) years, the proportion of men:women was 185:79, and 44 patients had sustained penetrating trauma.

**Table 2:** Overall characteristics of the eligible patients

Age	50 [33–65]
Sex (male/female)	185/79
Blunt/Penetrating trauma	220/44
SBP (mmHg)	102 [78.5–127]
RR (breaths/min)	25 [20–30.3]
GCS	14 [8–15]
RTS	7.55 [5.32–7.84]
ISS	25 [13–38]
PRBC (unit)^a^	16 [8–28]
FFP (unit)^a^	16 [9.5–26]
TRISS	0.701
TRAIS	0.076
Observed survival rate	0.776 (204/264)*

*SBP* systolic blood pressure, *RR* respiratory rate, *GCS* Glasgow coma scale, *RTS* revised trauma score, *ISS* injury severity score, *PRBC* packed red blood cells, *FFP* fresh frozen plasma, *TRISS* trauma and injury severity score, *TRAIS* TRISS adjusted increment in survivability

* *p* < 0.001 vs. mean probability of survival (Ps)

^a^ *n* = 152 (excluding patients who did not receive transfusion within 24 h or those with no detailed transfusion data)

The surgical procedures were as follows. Resuscitative thoracotomy to control hemorrhage, or to clamp the descending aorta (*n* = 63); clamshell thoracotomy simultaneously applied to repair thoracic organ injury (*n* = 14), and aortic cross clamping via left thoracotomy for hemorrhage control (*n* = 29, with abdominal organ injury or pelvic fracture). Celiotomy was performed for abdominal organ injury in 214 patients and retroperitoneal packing was carried out for severe pelvic fracture in 18 patients. The average time between arrival at the hospital and the operation was 7.1 min in URS group. Unfortunately, total resuscitation volume before surgery and time between arrival at hospital and surgery in the OR were not able to be collected sufficiently because of missing data.

### Evaluation of survival rate

**Fig. 1:**
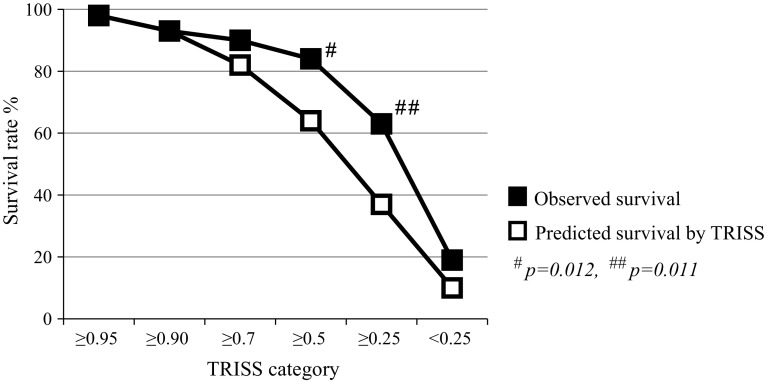
Observed survival rates in each Trauma and Injury Severity Score (TRISS) category: ≥0.95 (*n* = 103), ≥0.9 (*n* = 28), ≥0.7 (*n* = 40), ≥0.5 (*n* = 18), ≥0.25 (*n* = 27), and <0.25 (*n* = 48)

The overall survival rate of this cohort was significantly higher than the mean predicted survival rate (0.776 vs. 0.701; *p* < 0.001). To evaluate our performance of urgent hemostatic surgery in greater detail, the patients were classified into six TRISS categories (≥0.95, ≥0.9, ≥0.7, ≥0.5, ≥0.25, and <0.25). Figure 1 shows that the observed survival rate was significantly higher than the mean Ps between the TRISS category of 0.25 and less than 0.5 (0.630 vs. 0.365; *p* = 0.011) and that between 0.5 and less than 0.7 (0.833 vs. 0.603; *p* = 0.012).

In comparing the URS group (*n* = 97) and the non-URS group (*n* = 167), into which the eligible patients were divided, the RTS on hospital arrival was significantly lower in the URS group than in the non-URS group, reflecting the application of the URS policy. Furthermore, the URS group patients had more severe injuries not only anatomically, but also physiologically, particularly with respect to systolic blood pressure and consciousness (Table 3). With regard to the surgical procedure, celiotomy was performed significantly more frequently in the non-URS group (158/167, *p* < 0.001) than in the URS group (56/97), and other resuscitative procedures, including resuscitative thoracotomy (61 in the URS group vs. 2 in the non-URS group), retroperitoneal packing (17 vs. 1), and DCS (57 vs. 19), were carried out significantly more frequently in the URS group (*p* < 0.001).

**Table 3:** Characteristics of the urgent resuscitative surgery (URS) and non-URS group patients

	URS group (*n* = 97)	Non-URS group (*n* = 167)	*p* value
Age	54.5 [37.5–71.3]	48 [32–63]	0.001
Sex (male/female)	69/28	116/51	0.889
Blunt/penetrating trauma	88/9	132/35	0.016
SBP (mmHg)	72 [57.5–98]	118 [98–134]	<0.001
RR (breaths/min)	30 [20–35]	25 [20–30]	0.113
GCS	7 [3–13]	14 [13–15]	<0.001
RTS	5.03 [2.63–6.28]	7.84 [6.90–7.84]	<0.001
ISS	37 [25–43]	17 [10–26]	<0.001
PRBC (unit)^a^	26.5 [8–28]	10 [5.5–20]	<0.001
FFP (unit)^a^	23 [15–36.5]	10 [6–20]	<0.001
TRISS	0.402	0.875	<0.001
Observed survival rate	0.485 (47/97)*	0.946 (158/167)**	
Unexpected survival	18 cases (18.6%)	8 cases (4.8%)	<0.001

*SBP* systolic blood pressure, *RR* respiratory rate, *GCS* Glasgow coma scale, *RTS* revised trauma score, *ISS* Injury severity score, *TRISS* Trauma and injury severity score, *PRBC* packed red blood cells, *FFP* fresh frozen plasma

* *p* = 0.038, ** *p* < 0.001 vs. each TRISS value

^a^ *n* = 64 in URS and *n* = 88 in non-URS (excluding patients who did not receive transfusion within 24 h or those with no detailed transfusion data)

As mentioned above, the RTS was significantly lower in the URS group than in the non-URS group [5.03 (2.63–6.28) vs. 7.84 (6.90–7.84); *p* < 0.001]. Systolic blood pressure (SBP) and the Glasgow Coma Scale (GCS) score on ED admission were also significantly lower in the URS group than in the non-URS group. The ISS was significantly higher in the URS group than in the non-URS group [37 (25–43) vs. 17 (10–26); *p* < 0.001; Table 3]. The mean head AIS of the URS and non-URS groups was 1.54 and 0.9, respectively (*p* < 0.001). The observed survival rate was significantly higher than the predicted survival rate in the URS group (48.5 vs. 40.2%; *p* = 0.038), and a significant difference was also noted in the non-URS group (94.6 vs. 87.5%; *p* < 0.001). Furthermore, 18 patients in the URS group (18.6%) survived unexpectedly despite a *P*s score <0.5 vs. 8 in the non-URS group (4.8%) (*p* < 0.001).

Attempting to clarify the conditions in the URS group in further detail, patients with a SBP <90 mmHg had a significantly higher survival rate than that predicted by TRISS (0.433 vs. 0.309, *p* = 0.008) and those with a GCS score ≥9 also had a significantly higher survival rate than the predicted rate (0.795 vs. 0.681, *p* = 0.004). Among 86 patients in whom DCS was attempted, 56 for whom DCS was completely accomplished had a significantly higher survival rate than their predicted rate (0.614 vs. 0.423, *p* = 0.001) (Table 4). Multiple regression analysis showed that although each hemostatic procedure was not a significant predictor of mortality, the use of DCS as the hemostatic strategy in these 57 patients was found to be a significant predictor of survival (odds ratio 5.23, 95% confidence interval 0.113–0.526; *p* < 0.010).

**Table 4:** Outcomes according to specific subgroups

	Obs. surv.	TRISS	TRAIS	*p* value
Blunt injury (*n* = 17)	0.366	0.443	0.077	0.077
SBP <90 mmHg (*n* = 67)	0.433	0.309	0.124	0.008
SBP ≥90 mmHg (*n* = 30)	0.600	0.610	−0.009	0.900
GCS <8 (*n* = 53)	0.226	0.170	0.057	0.320
GCS ≥9 (*n* = 44)	0.795	0.681	0.114	0.004
RT (+) (*n* = 61)	0.328	0.293	−0.004	0.485
CT (+) (*n* = 13)	0.538	0.360	0.178	0.231
PP (+) (*n* = 17)	0.294	0.276	0.018	0.866
DCS (+) (*n* = 57)	0.614	0.423	0.191	0.001

*SBP* systolic blood pressure, *GCS* Glasgow coma scale, *RT* resuscitative thoracotomy, *CT* clamshell thoracotomy, *PP* pelvic packing, *DCS* damage control surgery, *TRISS* Trauma and injury Severity Score, *TRAIS* TRISS adjusted increment in survivability, *Obs. Surv.* observed survival

## Discussion

The aim of this study was to investigate the validity of and indications for our institution’s URS policy. To reflect the actual clinical situation, the parameters we selected for this study were limited to those readily available and able to be judged quickly. Regarding the patients’ vital signs contributing to the application of the URS policy, when the patient’s SBP was less than 90 mmHg or their GCS score was more than 9, the actual survival rates exceeded the calculated predictive values significantly. This indicates that application of the URS policy is appropriate when the patient does not have a serious initial head injury or has not yet fallen into deep unconsciousness as a result of hemorrhagic shock.

Importantly, it was revealed that performing DCS as the hemostatic strategy improved survival under these conditions. Approximately, 90% of our URS group patients underwent DCS because of their physiological and anatomical conditions. Damage control was accomplished in 57 patients, as revealed by the multiple regression analysis that the DCS implementation could improve the survival rate by five times. Various indications and interventions for implementing DCS in a scoping view have been reported [17, 18]. In this study, DCS was carried out based on our original indications [6], with several surgical interventions applied for hemorrhage control. A superiority of resuscitative thoracotomy vs. pelvic packing was not revealed, but the success or failure of DCS implementation formed the basis of our URS policy.

Comparing the cases of the same severity in the URS group and the non-URS group is theoretically possible, but not realistic if a patient with poor vital signs is treated under the non-URS policy. Hence, the URS strategy was applied absolutely, based on the differences in vital signs between the two groups, provided the conditions allowed for application of the URS policy. The strong points of this study were not to compare the two groups, but to compare the actual survival rate with the predictive rate by the *t* test for the URS group.

The present findings suggest the potential effectiveness of urgent resuscitative hemostatic surgery in the ED for patients with severe torso trauma, whose worsening vital signs render timely transport to the OR difficult. To achieve this, we established a protocol of rules and suitable equipment preparation, which make it possible to start URS within 3 min of the patient arriving at the ED. These preparations include the immediate availability of surgical and other instruments for urgent thoracotomy and laparotomy, external fixation for an unstable pelvic fracture, a rapid infuser device for DCR, and a protocol for massive transfusion.

To standardize the initial management of trauma patients, the Japan Advanced Trauma Evaluation and Care (JATEC) program was established for surgeons and emergency physicians in 2003. This was modified based on Advanced Trauma Life Support to suit various medical situations in Japan. It was designed to decrease “preventable trauma death” caused by the lack of basic trauma management, for example, surgically securing the airway for airway obstruction, tube thoracostomy for tension pneumothorax, or appropriate fluid resuscitation for hemorrhagic shock. Hondo et al. reported that trauma education through JATEC has succeeded in decreasing in-hospital trauma mortality, based on 96,664 patients registered in the Japan Trauma Data Bank [19]. However, they noted that mortality was significantly worse for severely injured patients or those requiring surgical procedures. This revealed that the JATEC is focused only on “basic trauma care”, because the contents of the JATEC are extremely basic concepts and skills for trauma and it does not guarantee saving patients with life-threatening injuries.

Many textbooks state that trauma care consists of “Primary survey and Resuscitation”, “Secondary survey”, and “Definitive treatment”. When hemodynamic stability is achieved by simple initial fluid resuscitation, repair of the injured organ is then recognized as “Definitive treatment” after “Secondary survey”. However, it is important that trauma resuscitation is conducted while keeping in mind the concept of DCR, which should include not only fluid resuscitation and massive transfusion, but also simultaneous hemostatic surgery to stabilize the patient’s hemodynamic conditions. For patients in severe hemorrhagic shock with impending cardiac arrest, although hemostatic surgery must be considered in the “Primary survey” section and not under “Definitive treatment”, JATEC does not emphasize this concept. These facts mislead many physicians to inappropriately consider that any surgical procedure, including hemostatic intervention, is “Definitive treatment”. As a result, many preventable trauma deaths after prolonged hemorrhagic shock still occur in Japan.

To prevent cardiac arrest and maintain vital signs while performing immediate hemorrhage control, we follow three strategies for trauma resuscitation in our institution: immediate surgical bleeding control, prompt decision to perform DCS, and application of the protocol for fluid resuscitation and massive transfusion (“seven bundle approaches”). During immediate hemostasis of the injured site, we have aggressively adopted resuscitative thoracotomy to achieve two goals [1]. The first is hemorrhage control by cross clamping of the descending aorta via left thoracotomy, especially for subdiaphragmatic injury. Cerebral and coronary blood flow can be maintained after aortic cross clamping even during active hemorrhage. The second is direct repair of chest injury accompanying hemostasis, such as performing clamshell thoracotomy to obtain a wide surgical field [20].

Basing the decision to perform DCS as the best hemostatic strategy on the classical criteria of the deadly triad, as outlined in the textbooks [21], would result in too long a delay for patients with severe torso trauma. We reported previously that the mortality of patients with severe abdominal trauma was 75% with significant differences, when the decision to perform DCS was based on three simple parameters (SBP <90 mmHg, base deficit >7.5 mmol/L, and a core temperature <35.5 °C) at the start of hemostatic surgery. Our results suggest that surgeons should initiate DCS when only one or two parameters are met and not wait for all three criteria [6]. While performing hemostatic surgery, systemic management should also be carried out according to the “Seven Tactics” for DCR [7] by non-surgical staff as follows: (a) application of the massive transfusion protocol (MTP) [8, 9, 22, 23], (b) permissive hypotension (SBP < 70–90 mmHg), (c) administration of noradrenaline [24], (d) calcium supplementation (>1.0 mmol/dL), (e) early administration of tranexamic acid [25], (f) aggressive rewarming, and (g) evaluation of the circulating volume using the pulse contour cardiac output measurement system (PiCCO) after resuscitation in the intensive care unit. Although Umemura et al. reported that fibrinogen and base excess levels can be used as an independent predictor for MTP [26], our MTP was achieved completely under the same indications. Our “seven tactics” significantly reduced 30-day mortality by 73% compared with non-bundle approaches (*p* < 0.01).

Immediate surgical bleeding control is a crucial part of trauma resuscitation in the three strategies mentioned above; however, physicians abide by the dogmatic custom that “the surgical operation must be done in the OR”. This custom can sometimes result in cardiac arrest or uncontrollable coagulopathy by the time the patient reaches the OR, because surgery in the OR needs completion of several unavoidable hospital procedures. Specifically, although it is advisable that surgical bleeding control be performed in a fully equipped OR, the reality is that in hospitals, such patients cannot be transported to the OR, which is typically some distance from the ED, as their vital signs deteriorate with every second. Even when the patient reaches the entrance of the OR without suffering a cardiac arrest, paperwork has to be completed to check the patient’s identification, the planned procedure, information on infections, and so on, for medical safety or risk management. The patient must then be moved onto the operating table, with some complicated processes for setting up multiple venous lines and monitoring leads. Establishing these may take longer than the surgeon expects, delaying the hemostatic operation further. Moreover, the OR may not be available because of the many scheduled operations that day. Such a delay in hemostasis greatly increases the risk of mortality of patients with severe trauma. Barbosa et al. [27] reported that delays in the time to operation for trauma patients who require emergent laparotomy lead to higher mortality.

Many US studies have reported that direct transfer to the operating room (DOR), bypassing the ED, for resuscitation increases the survival of severely injured patients [3–5, 28–30]. Fischer et al. reported in the 1970s that DOR improved the care of trauma patients and that it was a simple, economically feasible plan [30]. Rhodes et al. reported that non-arrested, hypotensive victims of blunt trauma requiring DOR for therapeutic laparotomy had higher than predicted survival (observed survival = 0.75 vs. average predicted survival by TRISS = 0.55) [5]. In a study conducted from 2010 onwards, Martin et al. described that with DOR, mortality was significantly lower than predicted (5 vs. 10%), concluding that the triage criteria for DOR included penetrating truncal injury, hypotension, and a GCS score <9 [2]. However, the DOR policy is not generally accepted by Japanese ORs for several reasons, including paperwork and rules. From another point of view, a hybrid ED, which comprises a fully prepared operating space with interventional radiology equipment in the ED, has recently been set up in many new or rebuilt hospitals, without applying the DOR policy [31]. Kirkpatrick and his co-workers reported that a hybrid OR with the same concept would provide new combined treatment paradigms [32]. However, as a hybrid facility depends greatly on the special layout or design, it would be impossible to immediately remodel our ED to be hybridized. Therefore, we modified the DOR concept regardless of the presence of a hybrid ED and established the URS policy, which involves performing hemostatic surgery in the ED, for patients with severe torso trauma. The potentially excess mortality could be avoided by the application of this policy, as evidenced in the present report. This might be because the URS policy closely resembles the DOR strategy and does not depend on the hospital design and layout. Our results indicate that URS is a suitable measure under “Primary survey”, which eliminates the delay and obstacles involved with patient transportation to the OR. For patients with life-threatening torso injury, if bleeding can be successfully controlled before cardiac arrest or coagulopathy in the prehospital setting, or during resuscitation in the ED, this would undoubtedly improve survival [1]. When hemostatic control needs to be achieved immediately upon arrival of the patient to the ED, the URS policy will support the achievement of this crucial goal.

Of all the patient’s vital signs on ED admission, the SBP was the most likely determinant of the URS policy application. However, the URS was not performed for 22 of 264 eligible patients in spite of an SBP <90 mmHg, because their SBP rose temporarily following the initial fluid resuscitation or hemodynamic stabilization after interventional radiology for unstable pelvic fracture, and they could be transported to the OR. Four of these twenty-two non-URS patients died; three of uncontrollable hemorrhage, and one of severe burns. Considering the validity of our URS policy, it was agreed that these 22 patients were put at additional hemorrhage risk by the excess fluid resuscitation to raise their blood pressure followed by severe bleeding.

This study has some limitations. First, it was a retrospective and single-institute investigation. The number of trauma cases in our facility was not small; however, the patient volume may be not sufficient for this type of cohort study. There was also no reality to conduct a randomized control trial to apply either the URS or the non-URS procedures for life-threatening cases. Second, although the decision about whether the patient should undergo hemostatic surgery in the ED (URS) or be transported to the OR was based on the criteria for the URS policy, the final decision depended on the experienced and skilled practitioner who treated the patient initially. Third, it did not investigate how many of the patients who were able to be transported to the OR were in the URS group. Moreover, we could not analyze the estimated time or the actual time from injury to the OR in the non-URS group. Although the collection of these data was possible, they were not recorded specifically for this study, so were not evaluated because of their low reliability. It cannot be denied that the presence of head injury affected outcomes in the URS group because of the higher mean AIS score compared with that in the non-URS group. Thus, further studies that completely eliminate the influence of head injury are necessary. Finally, as our facility is one of the base hospitals practicing the HEMS system, readers may have great concern about the influence of HEMS intervention on the result of our URS strategy [6]. To evaluate this influence, many parameters should be analyzed, including injury severity, injured sites, elapsed times, ability of onboarding physician, and interventions at the scene. These are complicated elements to include in judging the effectiveness of the URS policy. Therefore, we focused on only the patients’ vital signs on arrival to the hospital, disregarding these elements of the HEMS system.

In conclusion, while it is desirable to perform surgery in a fully equipped OR, urgent hemostatic operations in the emergency department must not be delayed if the patient’s vital signs are likely to deteriorate with transportation to the OR or during OR preparation and admission. Even when a patient with deteriorating vital signs can be transferred to the OR, the decision to initiate URS should not be delayed. As our URS concept achieved satisfactory survival outcomes for patients with severe torso trauma who required immediate hemostatic surgery, we consider that the best indications for application of the URS policy are an SBP <90 mmHg and a GCS ≥9 on ED arrival, and/or the need for DCS. Therefore, establishing appropriate criteria for deciding whether to perform hemostatic surgery in the ED or in the OR, regardless of the layout and facility design of hospitals, is a priority.
